# Prevalence and assessment of risk factors of chronic kidney disease in the ASIR region of Saudi Arabia

**DOI:** 10.1097/MS9.0000000000001755

**Published:** 2024-01-26

**Authors:** Mona Alshahrani, Laila Zawan Almalki, Noura Abdullah Hasoosah, Alhanouf Hussein Alahmari, Alya Musfer Alqahtani, Amjad Ali Alqahtani, Afrah Mohammed AL Muarfaj, Lamia Saeed Al Ghaseb, Faten Saad Alkahtani, Raghd Yahya Alsharif

**Affiliations:** a Faculty of Medicine, King Khalid University, Abha; b College of Medicine, King Khalid University, Abha, Saudi Arabia

**Keywords:** CKD, comorbidity, hypertension, NSAID, obstructive sleep apnoea, risk factor

## Abstract

**Introduction:**

Chronic kidney disease (CKD) is a major health concern in Saudi Arabia. The prevalence of CKD and associated risk factors in Saudi Arabia’s general population are not efficiently studied. The goal of this research is to determine the prevalence rate of CKD in the ASIR region and its associated epidemiological risk factors.

**Methodology:**

This is a cross-sectional study that used a comprehensive direct questionnaire to collect data on CKD prevalence and risk factors in the ASIR region of Saudi Arabia. The study was carried out in different age groups. Demographic characteristics, presence of CKD, and associated factors such as a history of acute kidney disease, obstructive sleep apnoea, family history, history of non-steroid anti-inflammatory drugs (NSAID), smoking status, comorbidities, and history of periodontal diseases were asked. The survey tool was validated through a pilot study, and a statistical *p* value of 0.05 was considered significant.

**Results:**

A total of 967 participants were included in the study, with a CKD prevalence rate of 1.9%. Sex, history of acute kidney disease, obstructive sleep apnoea, family history of kidney disease, smoking, diabetes, peptic ulcer disease, hypertension and hyperlipidemia were commonly found to be associated with CKD. A binary logistic regression model was carried out to assess the risk factors for CKD. Sex, history of acute kidney disease, family history of kidney disease, obstructive sleep apnoea, smoking status, peptic ulcer, diabetes, hypertension, hyperlipidemia, hypertension and diabetes, two or more comorbidities and NSAID use had an increased risk of CKD.

**Conclusion:**

Sex, history of acute kidney disease, obstructive sleep apnoea, family history, smoking, diabetes, hypertension, peptic ulcer, hyperlipidemia, multi-comorbidity, and use of NSAIDs are all associated with an increased risk of CKD. The prevalence of CKD in this study is comparatively lower when compared to other regions in Saudi Arabia.

## Introduction

Highlights

- The study found a lower-than-average chronic kidney disease prevalence of 1.9% in Saudi Arabia’s ASIR region.
- A range of risk factors including gender, obstructive sleep apnoea, and multiple comorbidities were identified.
- The research offers key insights for targeted chronic kidney disease prevention and management.

Chronic kidney disease (CKD) is characterized by a reduction in kidney function or structural damage (or both) persisting for more than three months. Over recent decades, the global prevalence of CKD has been increasing, imposing a significant burden on the healthcare system. The estimated prevalence of CKD is 13.4% (11.7–15.1%)^1^. In Saudi Arabia, the prevalence of CKD in the young population is ~5.7%^2^. In 2017, there were around two million cases of CKD in Saudi Arabia, with 3818 deaths attributed to the disease^3^. CKD substantially contributes to the global burden of mortality and morbidity, particularly through its impact on the cardiovascular system and the progression to end-stage kidney disease (ESKD). Data available on the exact incidence and prevalence of ESKD, as well as its association with CKD, remains limited. CKD is often associated with high rates of cardiovascular comorbid diseases. Therefore, early detection of CKD through screening is crucial, as it can prevent the progression to ESKD in many individuals.

Diabetes, hypertension, and obesity are significant risk factors in the aetiology of CKD, and their prevalence is notably high in Saudi Arabia. In Gulf countries such as the United Arab Emirates, Saudi Arabia, Bahrain and Kuwait, the WHO reports a very high prevalence of diabetes^4^. It is estimated that 20–40% of individuals with diabetes will develop diabetic nephropathy during the latter stage of their illness. Consequently, as the number of diabetic patients increases, a corresponding rise in the incidence of CKD is anticipated^5^. Moreover, the prevalence of obesity, which has been increasing in the past two decades, is now considered an independent risk factor for the development and progression of CKD^6^. Hypertension represents a leading attributable cause of CKD and ESKD. A critical mechanism in this process is the increase in blood pressure in CKD patients, which leads to sodium retention and activation of the renin-angiotensin system^7^. Furthermore, non-steroidal anti-inflammatory drugs (NSAIDs) have been associated with acute kidney injury as well as disease progression in CKD^8,9^. Other factors impacting CKD include smoking^10^, family history of kidney diseases, heart rate, damage to the kidney, older age, race and sex^11^.

Research to determine the prevalence rate of CKD and its risk factors in Saudi Arabia, particularly in the ASIR region, is limited^4^. This research aims to identify the prevalence of chronic kidney disease and associated epidemiological risk factors.

## Methods

### Study design and population

This cross-sectional study was conducted to assess the prevalence and risk factors of CKD in the ASIR region of Saudi Arabia. A total of 967 participants were included, representing various demographics from across the region. The study encompassed individuals of different age groups visiting shopping malls in ASIR to ensure a diverse sample reflective of the general population.

The inclusion criteria for participants were residency in the ASIR region, willingness to participate, and the ability to provide relevant demographic and health information. The exclusion criteria were non-residency in the ASIR region, inability to provide informed consent or understand the study’s purpose, and submission of incomplete questionnaire data.

### Data collection and study outcomes

Based on the pre-determined factors, we developed a questionnaire of 14 items and conducted a reliability analysis. The survey included demographic information such as gender, marital status, nationality, and ethnicity. Furthermore, questions about the presence of CKD and associated factors such as a history of acute kidney disease, obstructive sleep apnoea, family history, history of NSAID, smoking status, comorbidities and history of periodontal diseases were included based on the literature^10,12–15^. The purpose of the survey was to estimate the prevalence of CKD and its associated risk factors. During the survey, participants were interviewed by data collectors. The team’s data collectors used Google Forms, which included an ethical statement on the participation of each individual.

### Sample size calculation

For the determination of the sample size, we utilized the Raosoft sample size calculator, a widely accepted tool for epidemiological studies. This calculation was based on the anticipated CKD prevalence in the ASIR region, the desired confidence level, and the margin of error. Given the estimated prevalence rate of CKD in Saudi Arabia, we set the confidence level at 95% with a 5% margin of error. This approach yielded the required sample size of 742 participants. However, to enhance the robustness of our findings and account for potential non-responses or incomplete data, we expanded the sample size to 967 participants.

### Statistical analysis

The collected data were analyzed using the Statistical Package for the Social Sciences (SPSS), version 26. The reliability of the questionnaire was assessed using Cronbach’s alpha, with values ranging from 0.6 to 0.8 considered acceptable and those from 0.8 to 1.00 regarded as very good^16^. Means and standard deviations (SD) were used to present continuous data, while frequencies and percentages were used to present categorical data. The χ^2^/Fisher’s exact test and the unpaired two-tailed Student’s *t*-test were utilized to analyze differences in participant characteristics and risk factors for CKD. A *p*-value of less than 0.05 was set for statistical significance. Additionally, a binary logistic regression was performed to estimate the unique relationship between the included variables and CKD status.

### Ethical statement

The study was approved by the institutional review board of King Khalid University with approval number: ECM#2023-1202. In accordance with the Declaration of Helsinki, this research was registered in the Research Registry with the registration number: researchregistry9629. The study followed the Strengthening the Reporting of Cohort, Cross-Sectional, and Case-Control Studies in Surgery (STROCSS) 2021 guidelines. A consent form was obtained from all participants for their responses to be included in the research for publication purposes. All consent forms were archived by the review board committee.

## Results

### Questionnaire reliability analysis

A 14-item questionnaire was used to investigate the prevalence and associated factors of CKD. The obtained Cronbach’s alpha for the questionnaire was 0.611. According to Pallant *et al*.^16^., Cronbach’s alpha in the range of 0.6–0.8 is moderate but acceptable (Table 1).

**Table 1:** Reliability analysis result.

Measure	No. items (*n*)	Cronbach’s alpha (α)	
CKD and associated factors	14	0.611	Moderate acceptable

CKD, chronic kidney disease.

### Study population

The study analyzed 967 responses from different regions of Saudi Arabia. Most of the participants were female (70.6%). The mean age of the participants was 33.93 ± 13.81 years, and the mean BMI was 25.99 ± 11.82 kg/m^2^. A history of acute kidney disease was reported in 66 (6.8%) of the participants, while obstructive sleep apnoea was found in 62 (6.4%) of the participants. Moreover, 154 (15.9%) were found to have a family history of kidney disease, while 12.9% of respondents were smokers. Lastly, a history of periodontal disease was reported in 16.0% of participants (Table 2).

**Table 2:** Socio-Demographic characteristics of participants

	Frequency, *n* (%)
Male	284 (29.4)
Female	683 (70.6)
Marital status	
Single	418 (43.2)
Married	485 (50.2)
Widowed	36 (3.7)
Divorced	28 (2.9)
Nationality	
Non-Saudi	37 (3.8)
Saudi	930 (96.2)
Ethnicity	
Asian	18 (1.8)
Black	19 (2)
Middle Eastern	863 (89.2)
White	67 (6.9)
Presence of CKD	
Yes	18 (1.9)
No	949 (98.1)
History of acute kidney disease	
Yes	66 (6.8)
No	901 (93.2)
Obstructive sleep apnoea	
Yes	62 (6.4)
No	905 (93.6)
Family history of kidney disease	
Yes	154 (15.9)
No	813 (84.1)
Smoker	
Yes	125 (12.9)
No	842 (87.1)
History of NAIDS use	
Yes	119 (12.3)
No	848 (87.7)
History of periodontal disease	
Yes	155 (16.0)
No	812 (84.0)
	Mean ± SD
Age	33.93 ± 13.81
Weight (Kg)	67.52 ± 16.15
Height (cm)	162.33 ± 10.04
BMI (Kg/M^2^)	25.99 ± 11.82

CKD, chronic kidney disease; NSAID, nonsteroid anti-inflammatory drug.

### Prevalence of CKD and associated factors

**Figure 1:**
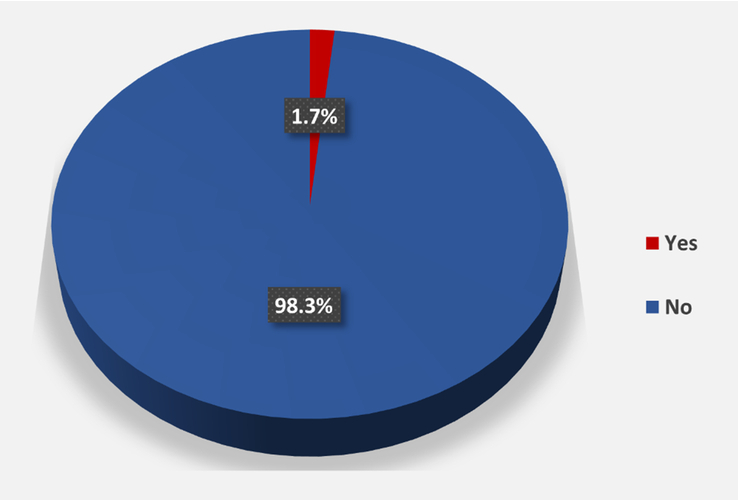
Distribution of responses based on the presence and absence of chronic kidney disease.

Among the respondents, 18 (1.9%) participants had been diagnosed with CKD (Fig. 1). Regarding the associated risk factors, we compared them with the presence of CKD. As a result, a statistical correlation was found in sex (*P* = 0.001), diabetes (*P*=0.004), hypertension (*P*<0.001), peptic ulcer disease (*P*=0.002), hyperlipidemia (*P*=0.028), hypertension and diabetes (*P*=0.002), more than two comorbidities (*P*<0.001), history of acute kidney disease (*P* < 0.001), obstructive sleep apnoea (*P* <0.001), family history of kidney disease (*P* = 0.004), smoking status (0.021) and history of NSAID use (*P* < 0.001). There was no significant difference observed in age and BMI between the participants with and without CKD (*P* = 0.325 and *P* = 0.686, respectively). Regarding those with CKD, it was found that most of the CKD-diagnosed participants were male (71.4%). Meanwhile, for those with comorbidities, seven (38.9%) CKD participants reported the presence of diabetes, 27.8% reported peptic ulcer disease, 55.6% of them had hypertension, 22.2% had hyperlipidemia, 27.8% had hypertension and diabetes, and 61.1% had two or more comorbidities. Over half of the CKD participants had a history of acute kidney disease, while 44.4% of them reported a family history of kidney disease. Lastly, obstructive sleep apnoea was reported in 61.1% of CKD participants, while around one-third (33.3%) were smokers (Table 3).

**Table 3:** Univariate analysis of factors in subjects with and without chronic kidney disease

	Presence of CKD		
	Yes (*n*=14), *n* (%)	No (*n* = 816), *n* (%)	*P*
Sex			
Male	13 (72.2)	271 (28.6)	**0.001**
Female	5 (27.8)	678 (71.4)	
Marital status			
Single	9 (50.0)	409 (43.1)	0.756
Married	9 (50.0)	476 (50.2)	
Widowed	0	36 (3.8)	
Divorced	0	28 (3)	
Nationality			
Non-Saudi	0	37 (3.9)	1.000
Saudi	18 (100)	912 (96.1)	
Ethnicity			
Asian	1 (0.0)	17 (1.8)	0.213
Black	1 (7.1)	18 (2)	
Middle Eastern	10 (50.0)	853 (89.6)	
White	6 (42.9)	61 (6.6)	
BMI			
Underweight	3 (17.6)	68 (7.2)	0.141
Normal	5 (29.4)	428 (45.1)	
Overweight	9 (52.9)	453 (47.7)	
Diabetes			
Yes	7 (38.9)	112 (11.8)	**0.004**
No	11 (61.1)	837 (88.2)	
Peptic ulcer disease			
Yes	5 (27.8)	48 (5.1)	**0.002**
No	13 (72.2)	901 (94.9)	
Hypertension			
Yes	10 (55.6)	96 (10.1)	**<0.001**
No	8 (44.4)	853 (89.9)	
Hyperlipidemia			
Yes	4 (22.2)	61 (6.4)	**0.028**
No	14 (77.8)	888 (93.6)	
Asthma			
Yes	0	11 (1.2)	1.000
No	18 (100)	938 (98.8)	
Diabetes and hypertension			
Yes	5 (27.8)	48 (5.1)	**0.002**
No	13 (72.2)	901 (94.9)	
Only one comorbidity			
Yes	1 (5.6)	22 (2.3)	0.354
No	17 (94.4)	927 (97.7)	
Two comorbidities			
Yes	11 (61.1)	34 (3.6)	**<0.001**
No	7 (38.9)	915 (96.4)	
History of acute kidney disease			
Yes	11 (61.1)	55 (5.8)	**<0.001**
No	7 (38.9)	894 (94.2)	
Obstructive sleep apnoea			
Yes	11 (61.1)	51 (5.4)	**<0.001**
No	7 (38.9)	898 (94.6)	
Family history of kidney disease			
Yes	8 (44.4)	146 (15.4)	**0.004**
No	10 (55.6)	803 (84.6)	
Smoker			
Yes	6 (33.3)	119 (12.5)	**0.021**
No	12 (66.7)	830 (87.5)	
History of NSAIDs use (regularly)			
Yes	9 (50.0)	110 (11.6)	**<0.001**
No	9 (50.0)	839 (88.4)	
Age	39.29 ± 22.10	33.84 ± 13.61	0.325
BMI	26.01 ± 11.90	24.84 ± 5.73	0.686

CKD, chronic kidney disease; NSAID, nonsteroid anti-inflammatory drug.

*P*-value <0.05 is statistically significant.

A binary logistic regression model was carried out to assess the risk factors of CKD. Sex [odds ratio (OR): 6.50 CI: 2.29–18.42], history of acute kidney disease (OR: 7.84 CI: 1.33–22.60), family history of kidney disease (OR: 4.40 CI: 1.70–11.33), obstructive sleep apnoea (OR: 11.20 CI: 4.16–30.11), smoking status (OR: 3.48 CI: 1.28–9.46), peptic ulcer (OR: 7.22 CI: 2.47–21.07), diabetes (OR: 4.75 CI: 1.80–12.51), hypertension (OR: 11.10 CI: 4.28–28.81), hyperlipidemia (OR: 4.15 CI: 1.32–13.01), hypertension and diabetes (OR: 7.22 CI: 2.27–21.07), two or more comorbidity (OR: 7.12 CI: 2.48–22.07) and NSAIDs use (OR: 5.48 CI: 1.70–11.33) have an increased risk of developing CKD (Table 4).

**Table 4:** Logistic regression analysis of factors associated with CKD

	Odds ratio (95% CI)	*P*
Sex (reference: female)	6.50 (2.29–18.42)	<0.001
History of acute kidney disease	7.84 (1.33–22.60)	<0.001
Obstructive sleep apnoea	11.20 (4.16–30.11)	<0.001
Family history of kidney disease	4.40 (1.70–11.33)	0.002
History of NSAIDs use (regularly)	5.48 (1.33–22.60)	0.001
Smoking status	3.487 (1.28–9.46)	0.014
Peptic ulcer	7.22 (2.47–21.07)	<0.001
Diabetes	4.75 (1.80–12.51)	0.002
Hypertension	11.10 (4.28–28.81)	<0.001
Hyperlipidemia	4.15 (1.32–13.01)	0.014
Hypertension and diabetes	7.22 (2.27–21.07)	<0.001
Two or more comorbidities	7.12 (2.48–22.07)	<0.001

CKD, chronic kidney disease, NSAID, nonsteroid anti-inflammatory drug.

## Discussion

Our study results indicate a higher distribution of female and Saudi participants, with a reported prevalence of 1.9% in our study population. This prevalence rate contrasts with previous studies in Saudi Arabia. For instance, Alsuwaida *et al*.^2^ reported a 5.7% prevalence rate in a community-based screening program in Riyadh, involving 491 young Saudi participants. Similarly, results of a comprehensive survey in 2014 conducted by Ahmed *et al*.^17^ in the Hail region among 2946 participants depicted a CKD prevalence rate of 9.4%. Another cross-sectional study in the Hail region (2012) reported a 7.8% CKD prevalence among 2800 participants, with a female population distribution of 56.2%^18^. Mousa and colleagues highlighted that over 20 000 people in Saudi Arabia are currently receiving dialysis, and 9810 kidney transplant recipients are under follow-up care. The projected age-standardized prevalence of CKD stages 1–5, excluding renal replacement treatment, is 9892 per 100 000 people in Saudi Arabia. These figures underscore the limited availability of studies on CKD epidemiology in the region.

The occurrence of ESKD in the Middle East, covering countries like Iran, Egypt, Turkey, Tunisia, Yemen, Syria, Lebanon, Qatar, and Iraq, varied from 55 to 818 per million population. Additionally, the prevalence of CKD stages 3–5 in Abu Dhabi was reported as 2.8% in females and 4.6% in males^19^.

One prevalent issue is the insufficient early identification of CKD and its associated risk factors. Often, CKD is inadequately diagnosed and treated in primary care settings. Diabetes-linked kidney disease is frequently underreported in the Middle East. Moreover, studies indicate a significant underdiagnosis (33%) and undertreatment (76%) of hypertension, a key risk factor, in the United Arab Emirates^20^.

Amouzegar *et al*.^21^ reported that CKD frequency ranged from 5.2% in Yemen to 10.6% in Iran among 11 Middle Eastern countries included in the survey. of the burden of CKD-associated deaths and disability-adjusted life-years was highest in Saudi Arabia and Jordan^21^. In a population-based study from the Middle East conducted in 2018, a cumulative incidence of 11.4% for stages 3–5 of CKD was observed over a 9-year period, with an incidence rate of 164.8 per 10 000 person-years for these disease stages^22^. Additionally, Naser *et al*.^23^ revealed in their meta-analysis that in patients with diabetes mellitus, the combined estimate of CKD prevalence was 28.96%. Notably, all these studies from the Gulf and Middle Eastern countries reported a prevalence higher than our findings.

Hill *et al*.^24^, in a 2016 meta-analysis, reported a global prevalence of CKD at 13.4%, with stages 3–5 showing a prevalence of 10.6%. The Global Burden Disease study in 2017 estimated a staggering 697.5 million CKD cases worldwide. Notably, China (132.3 million cases) and India (115.1 million cases) collectively accounted for almost a third of the global CKD burden. Moreover, over 10 million cases were reported in Bangladesh, Brazil, Indonesia, Japan, Mexico, Nigeria, Pakistan, Russia, the United States, and Vietnam combined. In 2017, more than 1 million prevalent cases of CKD were identified in 79 of the 195 nations covered, accounting for 9.1% of the world’s population^3^.

A cross-sectional study by Kibria and Crispen in the United States spanning different periods 2003–2006, 2007–2010, 2011–2014, and 2015–2018, showed age-adjusted CKD prevalence of 14.1% (13.1–15.0%), 13.0% (12.3–13.8%), 14.0% (13.0–15.1%) and 13.3% (12.3–14.4%), respectively^25^. Another cross-sectional study from the United States by Vart and colleagues in 2020 involving 54,554 participants indicated an increase in the prevalence of stage 3 and stage 4 CKD, adjusted for age, sex, and race/ethnicity, from 3.9% in 1988–1994 to 5.2% in 2003–2004, then stabilizing at 5.1% in 2015–2016. Race/ethnicity considerably affected the adjusted CKD prevalence trend in different ways^26^. In England, a longitudinal cohort study with 3207 participants revealed that without screening, ~44% of CKD cases remained undetected, particularly in individuals older than 60 years, where the prevalence of CKD stages 1–5 was 18.2%^27^. Our study reported a lower prevalence of CKD; however, the need for regular CKD screenings for prevention, early diagnosis, and management remains critical. The epidemiology of CKD varies significantly with sex, with previous studies indicating a higher prevalence in women. However, men are more likely to reach kidney failure sooner, making the male gender a predictor of kidney failure^28^. In our study, 71.4% of CKD-reported subjects were men, with males having a 6.50-fold higher risk of developing CKD. There are several explainable factors for the increased prevalence of CKD and associated kidney failure in the male population, such as an unhealthier lifestyle, a lack of habitual moderate exercise, the presence of late-night dinner, bedtime snacking^29^, and testosterone hormone levels^30^.

Several factors contribute to the increased prevalence of CKD and associated kidney failure in the male population, including an unhealthier lifestyle, lack of moderate exercise, late-night dining, and higher testosterone hormone levels. Obesity is an independent risk factor for CKD^31^. In Saudi Arabia, the prevalence of obesity, which is higher than the global average (35% vs. 13%), is attributed to the adoption of a westernized lifestyle. This trend is concerning, as it leads to a higher risk of non-communicable diseases, including CKD^32,33^. Our study supports these findings, with most participants being obese (BMI > 26 Kg/m²). The mean BMI of CKD patients was notably higher (26.01 ± 11.90) compared to non-CKD subjects (24.84 ± 5.73). Additionally, the prevalence of obesity, alongside hypertension and diabetes—key risk factors for CKD development is found to be higher in the Saudi population^20^ This underscores the importance of early CKD screening and prevention strategies. The link between obesity and CKD is further explained through the role of adipokines or adipocytokines, hormone-like peptides produced by adipose tissue. Leptin and adiponectin, in particular, are crucial in this context, illustrating the connection between obesity, hypertension, and chronic nephropathy^34^.

The intricate relationship between metabolic syndrome (MetS) and CKD unfolds as a complex interplay. CKD, a prevalent progressive condition, sees the intervention of MetS, marked by heightened oxidative stress and inflammation. Potential mechanisms of renal damage include insulin resistance (IR), oxidative stress, proinflammatory cytokines, connective tissue growth, profibrotic factors, microvascular injury, and renal ischaemia^35^. MetS, in its own narrative, is characterized by an imbalance between prooxidants and antioxidants, leading to an overproduction of reactive oxygen species (ROS) and nitrogen species (RNS), resulting in oxidative damage^36^.

The kidneys, abundant in mitochondria, become susceptible to oxidative stress, primarily from dysfunctional mitochondria producing high levels of ROS. An imbalance in mitochondrial ROS (mtROS) regulation triggers cellular signalling pathways, contributing to kidney diseases^37,38^. Substantial evidence suggests that mtROS play a key role in the progression of kidney diseases due to the high mitochondrial content in renal tissue. Disruptions in mitochondrial homoeostasis, bioenergetic changes, and organelle stress are crucial contributors to the onset of renal diseases^39^.

The plot thickens as diabetes enters the scene, revealing a bidirectional relationship. Elevated blood glucose levels contribute to inflammation and oxidative stress, fostering cardiovascular dysfunction. OS and chronic inflammation play roles in IR, impaired insulin secretion, prediabetes, and diabetes^40,41^. Observational studies spanning a decade highlight positive correlations between inflammation, endothelial dysfunction, OS parameters, and the risk of type 2 diabetes. However, the association between specific OS markers and diabetes risk diminishes when considering BMI^42^. In obese children aged 3–6 years, OS markers like malondialdehyde (MDA), superoxide dismutase (SOD), and 3-nitrotyrosine (3-NT) correlate positively with body mass^43^.

In Saudi Arabia, type 2 diabetes mellitus stands out as a significant risk factor for CKD. The disease affects the kidney’s glomeruli, leading to CKD and eventually ESRD^44^. Our findings align with previous research, showing higher odds of CKD among diabetic patients. The role of hyperlipidemia in the progression of kidney disease is increasingly recognized^45,46^. Our study also observed a significant association between hyperlipidemia and CKD.

Smoking, by reducing kidney blood flow, increases the risk of CKD and is an independent risk factor for the progression to ESKD^47–49^, as evidenced by 33.3% of our study population being smokers. Obstructive sleep apnoea, more prevalent in CKD patients (40%) than in the general population, emerges as a significant risk factor for CKD and possibly accelerates renal function loss^50^. Furthermore, it has been shown that the prevalence of obstructive sleep apnoea is further increased in the more advanced stages of CKD^51^. In our study, obstructive sleep apnoea was found to be a significant risk factor for CKD (OR: 11.20, CI: 4.16–30.11). This is consistent with recently acquired evidence^52,53^. However, obstructive sleep apnoea patients are usually also observed with various other concomitant conditions such as diabetes, hypertension, and obesity. These are also established risk factors for CKD^54,55^. Abuyassin *et al*.^53^, suggested in their study that there is possibly a bidirectional link between CKD and obstructive sleep apnoea via several possible pathogenic processes, which raises the prospect that both conditions could operate as risk factors for one another. Recently, there has been a lot of interest in the bidirectional association between sleep-disordered breathing and CKD. Some patients with obstructive sleep apnoea appear to lose kidney function, particularly those with cardio-metabolic comorbidities^56^.

Hypertension is both a cause and a symptom of CKD, and our study found a significant association between hypertension and CKD. From our study, it is also evident that hypertension is significantly associated with CKD patients. Systematic review and meta-analysis have indicated that it poses a slightly lower risk for CKD in women than in men^57^. A study conducted in sub-Saharan Africa noted that hypertension in CKD is found to be associated with patients of older age, female sex, obesity, and hyperuricemia^58^. Uncontrolled hypertension is a major factor in CKD progression and increased cardiovascular disease risk^59^. More than half of the CKD patients in our study reported hypertension. Multimorbidity in CKD patients is linked to poorer renal outcomes.

In a pilot study that included 299 Saudis, 64.3% of the patients with different stages of CKD were found to be hypertensive. As per Mahmoud and colleagues, hypertension raises the chance of death as well as the onset of CKD. According to their study, compared to patients with normal blood pressure, hypertensive patients had a three times higher risk of cardiovascular death and four times higher risk of developing CKD^60^. Together, hypertension and diabetes are reported to account for 70% of ESRD^61^. In our study, we observed that 27.8% of CKD patients had both hypertension and diabetes. Also, from prior studies, it is evident that multi-comorbidity impacts the renal outcome in stage III–IV CKD. Lee *et al*.^62^ reported poor renal outcomes in CKD patients with multimorbidity. This is in line with our study results, which show that over half of the CKD patients reported more than two comorbidities.

Few have discussed the impact of NSAID use on the risk of CKD, particularly in patients with hypertension. A study from the Saudi Arabian province of Hail reported that 10.7% of the population regularly takes NSAIDs. These drugs are associated with both acute renal injury in the general population and the progression of disease in CKD patients^9^. Earlier research suggests that regular use of strong NSAIDs may increase the risk of developing CKD^63^. A propensity score-matched cohort study in Taiwan investigated the association between NSAIDs and CKD development in individuals with hypertension. The study found that NSAID use was associated with a 1.18-fold increased risk of CKD for usage between 1 to 89 days and a 1.32-fold higher risk for those taking them for more than 90 days in hypertensive patients^15^. Our results support the evidence that NSAID use is linked to an increased risk of CKD, necessitating close monitoring, particularly in hypertensive patients who are more susceptible to CKD. Adverse renal effects of NSAIDs include acute renal failure, nephrotic syndrome with interstitial nephritis, and chronic renal failure, which may involve glomerulopathy, interstitial nephritis, and papillary necrosis^64^.

The narrative weaves together the threads of MetS, CKD, and associated risk factors, offering a nuanced understanding of the challenges in preserving kidney health. The multifactorial nature of CKD, influenced by diabetes, hyperlipidemia, smoking, and obstructive sleep apnoea, underscores the need for comprehensive approaches to address and mitigate these interconnected risk factors. By unravelling this complex storyline, we pave the way for targeted interventions and strategies to prevent and manage CKD effectively.

### Strengths and limitations

This cross-sectional study in the ASIR region employs a comprehensive approach, utilizing a direct questionnaire to understand CKD prevalence and risk factors. The 14-item questionnaire underwent reliability analysis, ensuring robust data. Diverse age groups were included, and demographic information enhanced the study’s representativeness. Thorough exploration of CKD risk factors, ethical considerations, random sampling, and adherence to reporting guidelines bolster the study’s credibility.

However, data collection in shopping malls may introduce selection bias, and reliance on self-reported information poses challenges, potentially leading to underestimation. Findings may not generalize beyond the ASIR region, and the cross-sectional design limits causal inference. Social desirability bias and limited insights into CKD treatment are also notable considerations.

### Future research recommendations

To address limitations in our current study, future research should adopt a prospective approach within shopping malls, aiming to minimize selection bias and gather real-time data, thereby reducing reliance on self-reported information. A mixed-methods approach, combining quantitative data with qualitative methods, can offer a more comprehensive understanding of participants’ experiences and perceptions. To enhance data accuracy, supplementing survey findings with retrospective studies based on medical records is recommended. Furthermore, expanding study locations beyond the ASIR region will improve generalizability, providing a broader perspective on the topic. Consideration of longitudinal study designs can capture trends over time, contributing to a more robust exploration of causal relationships. Implementation of measures to mitigate social desirability bias, such as ensuring confidentiality and utilizing indirect questioning techniques, is crucial for obtaining more honest responses from participants. These adjustments will contribute to the validity and reliability of findings, providing a stronger foundation for future research.

## Conclusion

It may be concluded that male sex, history of acute kidney disease, obstructive sleep apnoea, family history, smoking, diabetes, hypertension, peptic ulcer, hyperlipidemia, multi-comorbidity, and use of NSAIDs are all associated with an increased risk of CKD. The prevalence of CKD in this study is comparatively lower than in previous investigations in Saudi Arabia. Extensive screening for high-risk individuals to diagnose CKD at an early stage and to follow more aggressive treatment methods for male patients as well as other important risk factors, especially hypertension, obstructive sleep apnoea, and patients on NSAIDs, is recommended.

## Ethical approval

The study was approved by the institutional review board of King Khalid University with approval number: ECM#2023-1202. A consent form was obtained from all participants for their responses to be included in the research for publication purposes. All consent forms were archived by the review board committee.

## Consent

Written informed consent was obtained from the patient for publication and any accompanying images. A copy of the written consent is available for review by the Editor-in-Chief of this journal on request.

## Source of funding

None.

## Author contribution

M.A. Conceived and designed the study, analyzed the data, and proofread the manuscript. Did not participate in data collection. L.A. was involved in study design, data collection, conceptualizing, writing, and proofreading. N.H. contributed to study design, data collection, conceptualizing, writing, and proofreading. A.A. participated in study design, data collection, conceptualizing, writing, and proofreading. A.A. engaged in study design, data collection, conceptualizing, writing, and proofreading. A.A. took part in study design, data collection, conceptualizing, writing, and proofreading. A.A.M. involved in study design, data collection, conceptualizing, writing, and proofreading. L.A.G. contributed to study design, data collection, conceptualizing, writing, and proofreading. F.A. participated in study design, data collection, conceptualizing, writing, and proofreading. R.A. engaged in study design, data collection, conceptualizing, writing, and proofreading.

## Conflicts of interest disclosure

The authors do not have any conflict of interest.

## Research registration unique identifying number (UIN)

The research was registered in Research Registry with registration number: researchregistry9629 https://www.researchregistry.com/browse-theregistry#home/registrationdetails/6534f9a13ee7200029b77d98/.

## Guarantor

None.

## Data availability statement

Data are available within the article.

## Provenance and peer review

Not commissioned, externally peer-reviewed.
